# Hypothalamic Regulation of Corticotropin-Releasing Factor under Stress and Stress Resilience

**DOI:** 10.3390/ijms222212242

**Published:** 2021-11-12

**Authors:** Kazunori Kageyama, Yasumasa Iwasaki, Makoto Daimon

**Affiliations:** 1 Department of Endocrinology and Metabolism, Hirosaki University Graduate School of Medicine, 5 Zaifu-cho, Hirosaki 036-8562, Aomori, Japan; mdaimon@hirosaki-u.ac.jp; 2 Department of Clinical Nutrition Management Nutrition Course, Faculty of Health Science, Suzuka University of Medical Science, 1001-1 Kishioka-cho, Suzuka 510-0293, Mie, Japan; iwasakiyasumasa@gmail.com

**Keywords:** glucocorticoid, hypothalamus, corticotropin-releasing factor, stress

## Abstract

This review addresses the molecular mechanisms of corticotropin-releasing factor (CRF) regulation in the hypothalamus under stress and stress resilience. CRF in the hypothalamus plays a central role in regulating the stress response. CRF stimulates adrenocorticotropic hormone (ACTH) release from the anterior pituitary. ACTH stimulates glucocorticoid secretion from the adrenal glands. Glucocorticoids are essential for stress coping, stress resilience, and homeostasis. The activated hypothalamic-pituitary-adrenal axis is suppressed by the negative feedback from glucocorticoids. Glucocorticoid-dependent repression of cAMP-stimulated *Crf* promoter activity is mediated by both the negative glucocorticoid response element and the serum response element. Conversely, the inducible cAMP-early repressor can suppress the stress response via inhibition of the cAMP-dependent *Crf* gene, as can the suppressor of cytokine signaling-3 in the hypothalamus. CRF receptor type 1 is mainly involved in a stress response, depression, anorexia, and seizure, while CRF receptor type 2 mediates “stress coping” mechanisms such as anxiolysis in the brain. Differential effects of FK506-binding immunophilins, FKBP4 and FKBP5, contribute to the efficiency of glucocorticoids under stress resilience. Together, a variety of factors contribute to stress resilience. All these factors would have the differential roles under stress resilience.

## 1. Introduction

**Figure 1:**
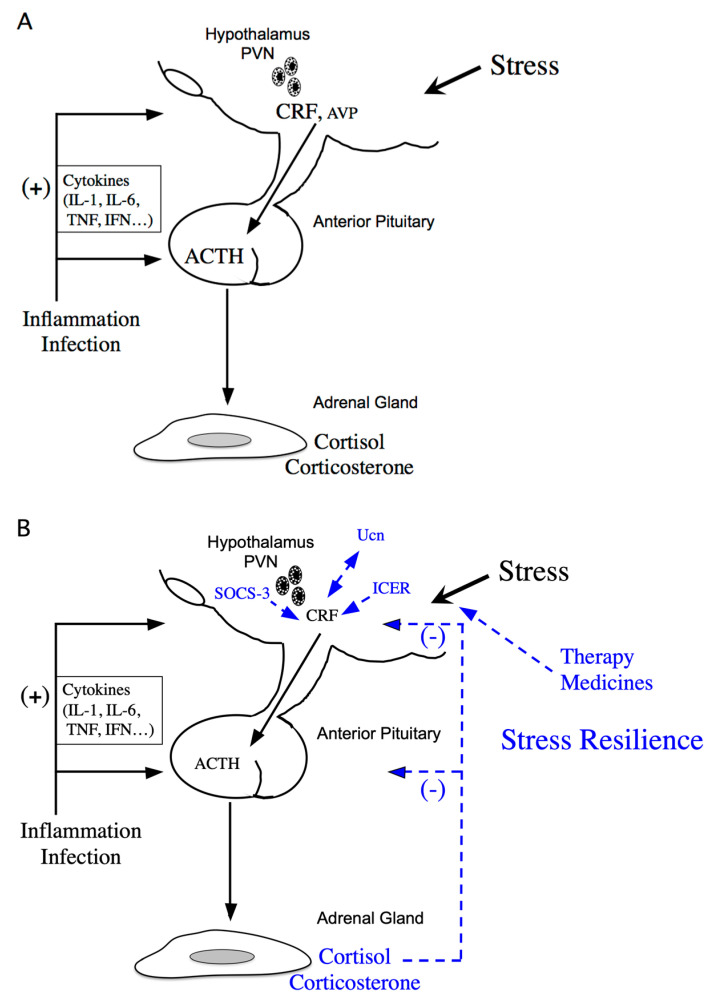
Schematic model of hypothalamic-pituitary-adrenal (HPA) axis regulation. (**A**) Activation of the HPA axis under stress. Corticotropin-releasing factor (CRF) plays a central role in controlling stress responses. Cytokines also stimulate CRF production under inflammation or infection. CRF, produced in the hypothalamic paraventricular nucleus (PVN), stimulates adrenocorticotropic hormone (ACTH) production from the corticotrophs of the anterior pituitary (AP). ACTH then stimulates corticosterone and cortisol release, the principal glucocorticoids in rodents and human, respectively, from the adrenal glands. (**B**) Regulation of the HPA axis under stress resilience. Glucocorticoids are produced by ACTH in the adrenal glands. Circulating glucocorticoids are critical for recovery from stress conditions. Both inducible cAMP-early repressor (ICER) and suppressor of cytokine signaling (SOCS-3) contribute to the negative regulation of CRF synthesis in the hypothalamus. The urocortin (Ucn)-CRF receptor type 2 mediates “stress coping” responses. Therapy or medicines also target this to alleviate stressed states.

Stress response is considered the physiological and behavioral response to internal or external stimulus. The stress response is adaptive, and stress resilience is regarded as the result of the adaptive response to a stressor [1,2]. The hypothalamic-pituitary-adrenal (HPA) axis is activated under various stressors (Figure 1A). Limbic structures, such as the central amygdala, bed nuclei of the stria terminalis, and nucleus accumbens shell of the extended amygdala, and the hippocampus, play an important role in stress responses. In parallel, stress responses also activate a physiological system for stress adaptation (Figure 1B).

Corticotropin-releasing factor (CRF) is a key hormone regulating the stress response, as it modulates the HPA axis [3]. CRF is produced in the hypothalamic paraventricular nucleus (PVN) in response to stress and plays an important role in the stress response as an initial activator of the HPA axis. CRF neurons in the PVN partially co-express arginine vasopressin (AVP), oxytocin, neurotensin, enkephalin, and cholecystokinin, and are primarily glutamatergic but partially gamma-aminobutyric acidergic (GABAergic) [4]: norepinephrine and glutamate stimulate CRF release under stress [5], while CRF is regulated by GABAergic inputs that inhibit its release [6]. CRF and AVP neurons in the parvocellular region of the PVN projecting to the external zone of the median eminence stimulate adrenocorticotropic hormone (ACTH) secretion from the anterior pituitary (AP) [7,8]. The CRF and AVP exert synergistic effects on ACTH secretion from the AP. CRF stimulates the synthesis and secretion of ACTH, encoded by the *propiomelanocortin* (*Pomc*) gene, in the AP [3] (Figure 1A). ACTH stimulates the secretion of corticosterone and cortisol, the main glucocorticoids in rodents and human, respectively, from the adrenal glands [9] (Figure 1A).

In *Crf* knockout (KO) mice, impaired adrenal responses have been shown in response to acute various stressors such as restraint, ether, and fasting [10,11,12]. The ACTH response to pain stress in *Crf* KO mice was also smaller than that in wild-type (WT) mice [13], suggesting a role of CRF on the increased ACTH release from the pituitary in response to stress. Two types of prohormone convertases contribute to *Pomc* processing. *Pomc* is cleaved to β-lipotropic hormone (β-LPH) and ACTH by the processing enzyme prohormone convertase-1. β-LPH and ACTH are then cleaved to β-endorphin (β-EP) and α-melanocyte stimulating hormone by the prohormone convertase-2, respectively. Pituitary *Pomc* mRNA levels are not attenuated [11,12]; however, ACTH contents are decreased in the AP of *Crf* KO mice [14], suggesting a role of CRF in *Pomc* processing. Immature *Pomc* is biologically inactive, and cannot stimulate cortisol production in the adrenal glands. Immunoreactive (ir) *β*-EP contents in the AP of *Crf* KO mice do not differ from those of WT mice. To determine the different molecular profile of *Pomc*-related peptides between WT mice and *Crf* KO mice, ir β-EP contents extracted from the AP of WT mice and *Crf* KO mice were assayed by gel filtration chromatography Gel filtration analyses, which revealed that a higher molecular weight form of ir β-EP, putative *Pomc*, increased in *Crf* KO mice, but the β-EP peak level was small and similar between the two groups [11]. These results suggest that CRF affects *Prohormone convertase-1* gene expression levels in the AP, thus modulating *Pomc* processing.

Cytokines are important mediators of the interaction between the neuroendocrine and immune systems, and inflammatory stress positively affects the HPA axis at multiple levels via cytokines. Following inflammatory challenges, interleukin (IL)-1, IL-6, tumor necrosis factor (TNF)-α, and interferon (IFN) stimulate CRF in the hypothalamus [15] (Figure 1A). For example, IL-6, a single 21- to 28-kDa glycoprotein produced in response to both immune activation and non-immune stress [16,17], increases *Crf* gene expression and secretion in the PVN [18,19]. Endogenous IL-1β and TNF-α productions are also involved in the stimulation of autocrine CRF production in hypothalamus [20,21]. HPA activation results in secretion of glucocorticoids. Glucocorticoids have pleiotropic effects on the immune system. Glucocorticoids inhibit inflammation, lymphocyte activation, and the production of B cells, T cells, and cytokines. Therefore, the bilateral interaction between the immune and endocrine system exists under in vivo stress response.

The suprachiasmatic nucleus (SCN) of the hypothalamus acts as the central circadian pacemaker. SCN neurons negatively regulate activity in CRF neurons in the PVN. As an additional role of CRF in the PVN, CRF neurons positively regulate orexin neurons, which promote wakefulness [22]. Therefore, circadian pacemaker regulates wakefulness via CRF neurons in the PVN. Stress may impair the circadian rhythmicity. Additionally, circadian clock disruption is also observed in many psychiatric disorders [23]. The disorders are accompanied by a reduced amplitude or altered phase in a wide range of rhythms, including cortisol, melatonin, and circadian clock genes. Dysfunction of the CRF system may contribute to the circadian clock disruption. On the other hand, disruption of the circadian clock is observed in obese mice [24]. Among the variety of factors contributing to stress resilience, ghrelin, an important regulator of metabolism or energy homeostasis, improves the disruption of the circadian rhythm in steatotic liver.

## 2. Molecular Mechanisms of CRF Regulation in the Hypothalamus

Many neurotransmitters and neuropeptides are involved in activation of CRF neurons in the hypothalamus. Serotonin, noradrenaline, neuromedin C, and thyrotropin-releasing hormone affect intracellular Ca^2+^ concentration in CRF neurons of the PVN [4] and activate CRF neurons. Pituitary adenylate cyclase-activating polypeptide (PACAP), a member of the secretin/glucagon/vasoactive intestinal peptide family, and glucagon-like peptide 1 (GLP1) also stimulate *Crf* gene activity in hypothalamic cells [25,26]. PACAP can contribute to CRF activation in the hypothalamus under emotional stress. In fact, the extended amygdala and the bed nuclei of the stria terminalis are identified as innervation sites of PACAP neurons. Both PACAP and the PACAP-selective PACAP receptor type 1 are highly expressed in the hypothalamus and the supraoptic nucleus [27,28]. PACAP increases *Crf* mRNA levels in the parvocellular region of the PVN, suggesting its involvement in the positive regulation of *Crf* gene expression [29]. The cyclic AMP (cAMP)-protein kinase A (PKA) pathway is involved in CRF synthesis by PACAP [25]. GLP1 also stimulates the activities of both *Crf* and *Avp* promoters in hypothalamic cells [26]. Basal promoter activities of *Crf* and *Avp* are increased in high glucose medium [26], while *Crf* and *Avp* promoter activities are increased by GLP1 in standard or low glucose medium but not in high. Hyperglycemia is, therefore, a stressor increasing the synthesis of CRF and AVP in the hypothalamus.

Ghrelin stimulates CRF release from rat hypothalamus explants, and ghrelin expression has been found in the rat hypothalamus [30]. Previous studies showed that ghrelin activated the HPA axis via CRF neurons of the PVN [30,31]. Glucocorticoids also stimulated the ghrelin-growth hormone-releasing peptide receptor type 1a (GHSR1a) system in hypothalamic cells. Dexamethasone increased *Ghrelin* mRNA levels, and *Ghsr1a* mRNA and protein levels. Finally, ghrelin increased *Crf* mRNA levels, as did dexamethasone, and both dexamethasone and ghrelin had an additive effect on *Crf* and *Ghrelin* mRNA levels [31,32].

Pyroglutamylated RFamide peptide (QRFP), an important regulator of metabolism and energy homeostasis, has orexigenic effects. QRFP acts via a specific receptor, G protein-coupled receptor (GPR) 103. QRFP might be related to stress behavior, because intense grooming is observed in response to acute QRFP administration in mice. *Gpr103* mRNA is expressed in the rat PVN. QRFP stimulates the HPA axis in vivo, and the *Crf* gene in hypothalamic cells [33]. QRFP stimulates the release of glucocorticoids, a representative orexigenic hormone, via CRF. Heat shock protein 70, a molecular chaperone produced under physical and environmental stress, also stimulates CRF in the hypothalamus, resulting in activation of the HPA axis [34].

The proximal *Crf* promoter shows several possible binding sites for transcriptional factors such as cAMP-response element (CRE), activator protein-1 (AP-1) protein binding sites, half glucocorticoid regulatory element (GRE), and half estrogen-responsive element (ERE) [35,36]. Forskolin or PACAP stimulates adenylate cyclase and then intracellular cAMP levels in hypothalamic cells [25]. Forskolin increases *Crf* transcriptional activity in hypothalamic and other cells [37,38,39]. Forskolin-induced *Crf* gene transcription is reduced in hypothalamic cells transfected with a mutant construct where the CRE element is mutated [40].

AP-1 proteins such as Fos and Jun are immediate-early gene products. The Fos protein family can dimerize with a Jun protein (Fos/Jun heterodimers), which binds to the regulatory sequences of target genes. *FosB* and *cJun* mRNA levels are regulated via PKA, protein kinase C (PKC), and Ca^2+^-dependent pathways. FosB or cJun overexpression potently increase *Crf* mRNA levels in hypothalamic cells, while their downregulation suppresses the stimulus-induced activation of *Crf* mRNA. Therefore, endogenous FosB and cJun are necessary for stimulus-induced *Crf* gene expression in hypothalamic cells [41]. The Fos/Jun heterodimer is involved in regulation of *Crf* gene expression. A truncated splice variant of FosB, FosB/ΔFosB was induced in the rat hypothalamus by surgical stress, and its expression upregulated by glucocorticoid removal. Induced FosB/ΔFosB expression was identified in CRH neurons of the PVN and AVP neurons of the supraoptic nucleus. In addition, forskolin-induced upregulation of *FosB* promoter activity was suppressed by glucocorticoids in the homologous hypothalamic cells. These results imply that glucocorticoids may be potent regulators of stress-induced *FosB* expression in hypothalamic neuroendocrine neurons [42].

The PKA pathway is mainly involved in cAMP-dependent CRF synthesis in the hypothalamus [43,44]. Activation of the PKA pathway leads to binding of CRE-binding protein (CREB) to the CRE on the *Crf* promoter in hypothalamic cells [45]. PKC and p38 mitogen-activated protein (MAP) kinase are also partially involved in the positive regulation of *Crf* gene expression in hypothalamic cells [40]. Thus, the PKA, PKC, and p38 MAP kinase pathways are all involved in *Crf* activation in hypothalamic cells. Meanwhile, glucocorticoids suppress cAMP-dependent increase in *Fos B* gene promoter activity [41].

Estrogen regulates the HPA axis by stimulating *Crf* gene expression in the hypothalamus, as shown when high levels of estrogen replacement increased basal *Crf* mRNA levels in the PVN of ovariectomized rats [46]. A physiologically relevant dose (10 nM) of estradiol (E2) stimulates both *Crf* gene transcription and mRNA expression in hypothalamic 4B cells [47]. This result suggests that the direct effect of E2 in increasing CRF in the PVN may contribute to the enhanced ACTH and cortisol levels observed in the midluteal phase. CRF neuron activation, in turn, may affect gonadal function in a bidirectional manner, because estrogens activate the HPA axis, while the estrogen-induced activation of CRF neurons would suppress gonadotropin-releasing hormone neurons. Moreover, E2 and diarylpropionitrile, an estrogen receptor β agonist, increase *Crf* transcriptional activity. Therefore, estrogen receptor β activation by estrogens induces *Crf* gene transcription in hypothalamic cells. Furthermore, treatment with both E2 and forskolin, a ubiquitous activator of adenylyl cyclase and cAMP, shows additive effects on *Crf* promoter activity [47]. Therefore, estrogens enhance *Crf* gene activation by cAMP under stress. Estrogen activates the HPA axis, and then activation of the HPA axis may suppress gonadotropin-releasing hormone.

As mentioned previously, stress resilience is considered the result of the adaptive response to a stressor. Circulating glucocorticoids are critical for recovery from stress conditions and essential for stress resilience (Figure 1B). Treatment with glucocorticoids in adrenalectomized rats does not perfectly prevent the increase in stress-induced *Crf* heteronuclear RNA [48]. Therefore, factors other than glucocorticoids might be involved in limiting CRF activation during stress. Inducible cAMP-early repressor (ICER), a cAMP-inducible member of the CRE modulator (CREM) family and a CREM repressor isoform, are such candidates [49,50]. CREM, CREB, and AP-1 bind to CRE promoter elements to stimulate transcription [51], while ICER acts as a competitive inhibitor of such CRE-dependent transcription [49]. Therefore, ICER can suppress the stress response via inhibition of the cAMP-dependent *Crf* gene [36] (Figure 1B). Suppressor of cytokine signaling (SOCS)-3 acts as a potent negative regulator of cytokine signaling [52]. SOCS-3 suppresses cytokine-induced ACTH production in corticotrophs [53]. IL-6 stimulates Janus kinase/signal transducers and activators of the transcription (JAK/STAT) signaling, while IL-6-induced SOCS-3 acts as a negative regulator and inhibits STAT phosphorylation by JAK at the receptor complex [54,55]. SOCS-3 is stimulated by IL-6 and cAMP, while SOCS-3 knockdown increases IL-6- or forskolin-induced *Crf* gene transcription, thereby contributing to the negative regulation of CRF synthesis in the hypothalamus [56] (Figure 1B).

## 3. Roles of the CRF Peptide Family and Stress-Related Peptides and Their Receptors under Stress

Urocortins (Ucns) are members of the CRF peptide family. Three Ucns have been found in mammals. Ucn1 has potent effects including appetite suppression and modulation of the cardiovascular system [57,58,59]. Ucn2 has more potent vasodilatory and cardiac inotropic effects than CRF and shows suppression of host resistance to infection via IL-10 upregulation [60]. Ucn3 modulates insulin secretion and Ca2+ influx in pancreatic β-cells [61] and improves cellular stress responses and glucose uptake [62].

**Figure 2:**
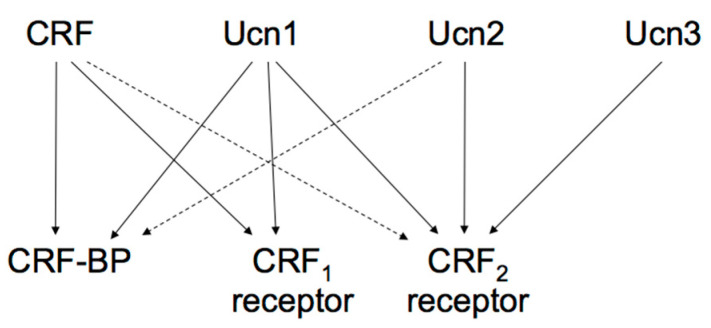
Proposed signaling mechanisms of corticotropin-releasing factor (CRF), urocortins (Ucns) and CRF receptors. CRF-BP, CRF-binding protein. Solid lines represent a high affinity binding, and dashed lines a low affinity binding.

The actions of the CRF family peptides are mediated by ≥ 2 distinct G protein-coupled receptors, namely the CRF receptor type 1 (CRF_1_ receptor) [63,64,65] and CRF receptor type 2 (CRF_2_ receptor) [66,67,68]. CRF has a higher affinity for the CRF_1_ receptor than for the CRF_2_ receptor (Figure 2) [69]. Ucn1 binds to both the CRF_1_ and CRF_2_ receptors, while Ucn2 and Ucn3 are highly selective for the CRF_2_ receptor, with little affinity for the CRF_1_ receptor (Figure 2) [57,70,71,72].

These two receptors share 69% amino acid homology [67] but have different tissue distributions and pharmacological properties with respect to ligands [69]. CRF_1_ receptor is mainly expressed in the pituitary, the brain, and various peripheral tissues. In pituitary corticotrophs, CRF_1_ receptor is the major subtype responsible for regulating ACTH synthesis and secretion. CRF_2_ receptor is located in different brain areas than the CRF_1_ receptor and is also abundant in the periphery. The CRF_2_ receptor has ≥ 3 alternative splice variants, CRF_2a_ receptor, CRF_2b_ receptor, and CRF_2g_ receptor. In the rat, CRF_2a_ receptor mRNA was found primarily in the brain and the pituitary [73,74]. In contrast, the CRF_2b_ receptor was predominantly expressed in peripheral sites such as the heart, gastrointestinal tract, and vascular smooth muscles [74]. CRF receptors primarily activate adenylyl cyclase and cAMP pathways through Gsα activation. In addition, CRF receptors can activate other types of Gα [75].

The CRF_1_ receptor is mainly involved in stress responses, depression, anorexia, and seizure, while the CRF_2_ receptor mediates “stress coping” responses such as anxiolysis in the brain [69], as shown by mice deficient in CRF_2_ receptor and the use of a CRF_2_ receptor antagonist, which display increased anxiety-like behaviors and a hypersensitive stress response [76]. Our previous findings also show that the intracellular signals mediated by the CRF_2_ receptor have an antagonistic effect to the CRF_1_ receptor. Therefore, specific CRF receptor subtypes may contribute to different stress responses and homeostasis. The lateral septum contributes to the control of stress response and anxiety. Activation of CRF_2_ receptor in the lateral septum and septohypothalamic circuits are reported to be involved in stress-induced persistent anxiety [77].

Hypothalamic CRF stimulates synthesis and secretion of ACTH via the CRF_1_ receptor in the anterior pituitary gland. *CRF_1_ receptor* mRNA levels are down-regulated by CRF via the cAMP-protein kinase A (PKA) pathway or by mRNA degradation via PKA [78]. Prolonged agonist activation of receptors leads to a loss of responsiveness or receptor desensitization. After agonist-activated stimulation of receptor signaling, the CRF_1_ receptor is down-regulated and desensitized in the pituitary corticotrophs. Generally, GPRs are desensitized by GPR kinases (GRKs). CRF desensitize the cAMP-dependent response by CRF_1_ receptors. GRK2 is involved in CRF-induced desensitization of the CRF_1_ receptor in corticotrophs, where the protein kinase A pathway plays an important role [79]. Intracellular molecules, such as β-arrestins, contribute to membrane receptor phosphorylation, desensitization, and trafficking of GPRs, thereby modulating ligand activated-receptor responses [80].

CRF-binding protein (CRF-BP), a 37-kDa secreted glycoprotein, binds to CRF and Ucn1 with an affinity equal to or greater than CRF receptors, suggesting its role as an important modulator of CRF, Ucns, and their receptors. CRF-BP inhibits CRF-induced ACTH secretion from pituitary corticotrophs and may show a similar CRF release action in the hypothalamus [81]. Potential CRF-BP roles might be modulating energy balance and feeding behavior. Additionally, changes in CRF-BP would relieve the action of CRF under stress. In humans, depression, Alzheimer’s disease, and inflammatory diseases have been related to CRF-BP dysregulation [81]. Investigating the potential roles and pathophysiology of CRF-BP may help elucidate the etiology of said diseases.

## 4. CRF Dysregulation and Therapy

CRF dysregulation in the hypothalamus and the HPA axis is associated with diseases related to stress and brain and inflammatory diseases. Hyperactivity of the HPA axis is found in patients with anxiety and mood disorders. Patients with posttraumatic stress disorder have decreased activity of the pituitary-adrenal axis, presumably due to exaggerated negative feedback or CRF hypersecretion with the subsequent down-regulation of the anterior pituitary CRF receptors [82]. Additionally, differences in CRF and the CRF_1_ receptor regulation may cause inherent differences in stress reactivity, which predicts susceptibility and resilience to a depressive phenotype [83,84].

The stressed state may be relieved or reduced via the HPA axis and other hormones by therapy, medicines or natural stimulation (Figure 1B). Successful therapy results in normal regulation of the HPA axis. For example, benzodiazepines reduce the activity of CRF neurons in the hypothalamus [85], tricyclic antidepressants and selective serotonin reuptake inhibitors partially act by modulating the HPA axis, while escitalopram, a selective serotonin reuptake inhibitor, inhibits CRF expression in the hypothalamus and hippocampus, increasing their glucocorticoid receptor (GR) expression. Both mineralocorticoid receptors and GRs in the PVN are involved in the regulation of depressive and anxiety-like behaviors [86]. The simultaneous restoration of both receptors and CRF in the PVN might help in the treatment of depression and anxiety. Nature, forests, urban green space, plants, and wooden materials reduce sympathetic nervous system activation and lower the blood pressure [87], and probably normalize the HPA axis. Oxytocin in the hypothalamus has been shown to facilitate affiliative social behaviors and to induce anxiolytic actions. For instance, post-weaning stroking procedures induce affiliative responses via activation of oxytocin neurons in the caudal PVN [88].

## 5. Negative CRF Feedback Mechanisms in the Hypothalamus

Glucocorticoids inhibit CRF production in the hypothalamic PVN and ACTH production in the AP, modulating its own production (Figure 1B). Simultaneously, glucocorticoids inhibit CRF neurons in the PVN by binding to GRs in the hippocampus [86].

**Figure 3:**
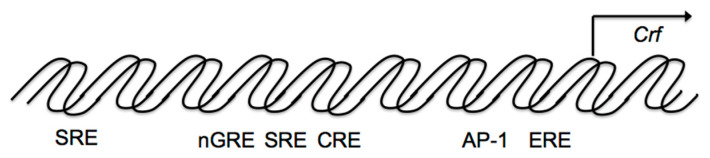
Schematic model of transcriptional regulation of the corticotropin-releasing factor (*Crf*) promoter. Possible binding sites for transcriptional factors such as the cAMP-response element (CRE), activator protein 1 (AP-1) protein (Fos/Jun) binding sites, the half glucocorticoid regulatory element (GRE), and the half estrogen-responsive element (ERE) in the proximal *Crf* promoter. The *Crf* promoter region also contributes to glucocorticoid inhibition as a negative GRE (nGRE), as it includes a serum response element (SRE).

The HPA axis is regulated by a negative feedback mechanism (Figure 1B). In fact, hypothalamic parvocellular neurons express GRs, and glucocorticoids negatively regulate *Crf* gene expression directly in the hypothalamus. The *Crf* promoter region contributes to inhibition by glucocorticoids by acting as a negative GRE (nGRE) (Figure 3), even though the *Crf* promoter does not contain a classical consensus GRE, there are several regions GRs can bind to [89,90]. Glucocorticoids can inhibit CREB and cFos in the PVN [91,92,93].

Additionally, other promoter regions are involved in the inhibition of *Crf* gene expression in hypothalamic cells (Figure 3). *Crf* promoter sequences between –248 and –233 bp are also involved in the glucocorticoid suppression of cAMP-stimulated *Crf* promoter activity (Figure 3), including a serum response element (SRE) [94], which contributes to the negative response to glucocorticoids, because the GR can bind to SRE and inhibit promoter activation by antagonizing positive transcription [94]. Therefore, in addition to nGRE, the glucocorticoid suppression of cAMP stimulated CRF promoter activity may also be caused by SRE in hypothalamic cells.

**Figure 4:**
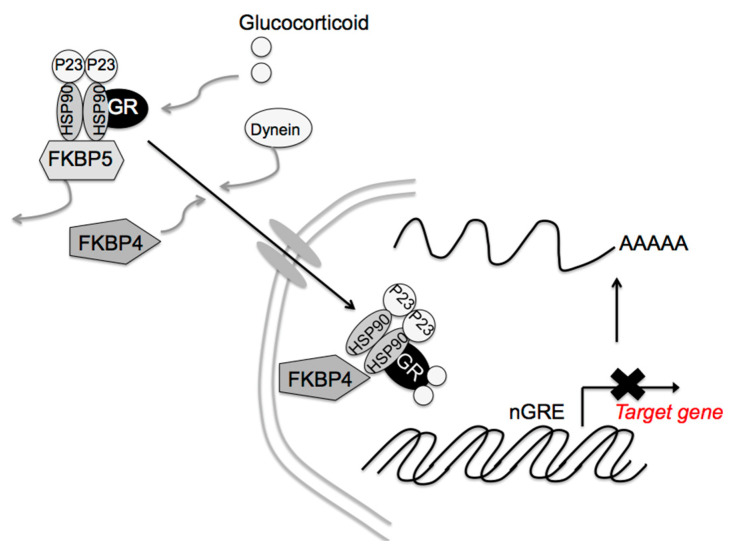
Proposed FK506-binding immunophilin 4 (FKBP4) and FKBP5 signaling mechanisms by glucocorticoids. After cortisol binding to the glucocorticoid receptor (GR), FKBP5 is replaced by FKBP4, resulting in complex translocation to the nucleus. Newly formed GR/heat shock protein 90 (HSP90)/FKBP4 complexes generally accumulate in the nucleus. The GR then modulates the target gene expression. [Modified from Ref. ([102], *Int. J. Mol. Sci.* **2021**, *22*, 5724.) with permission of the publisher.] Copyright 2021, MDPI.

Glucocorticoid signaling is mediated via the GR, 11β-hydroxysteroid dehydrogenases, and the FK506-binding immunophilins, FKBP52 (FKBP4) and FKBP51 (FKBP5) [95,96,97]. FKBP4 and FKBP5 differentially regulate dynein interactions and GR nuclear translocation, respectively [98]. In the absence of corticosterone, the GR is retained in the cytoplasm as a complex containing one GR molecule, heat shock protein (HSP) 90 dimer, HSP90-binding protein P23, and FKBP5 [99,100]. After glucocorticoid binding to the GR, FKBP5 is replaced by FKBP4, resulting in complex translocation to the nucleus (Figure 4). The GR then acts on *Crf* gene expression. Thus, FKBP4 contributes to the negative feedback effect of glucocorticoids. GR activity also stimulates *Fkbp5* gene transcription [100,101], the higher FKBP5 levels in turn inhibit GR translocation to the nucleus, diminishing the effects on glucocorticoids’ target genes. In fact, *Fkbp4* contributes to the negative feedback of glucocorticoids, and *Fkbp5* reduces the efficiency of the glucocorticoid effect on *Pomc* gene expression in pituitary corticotrophic cells [102]. *Fkbp5* deletion in the PVN dampens the acute stress response and increases GR sensitivity [103], while its overexpression in the PVN produces chronic overactivation of the HPA axis [103]. That way, the differential effects of FKBP4 and FKBP5 in the PVN contribute to glucocorticoid efficiency and stress resilience. Other molecules, such as HSP90, HSP90-binding protein P23, and GR itself may be involved in these glucocorticoid effects. Post-translational regulation of these molecules, such as protein phosphorylation, acetylation, and SUMOylation, as well as expression regulation, may also contribute to said effects.

Additionally, Itoi et al. performed genome-wide analysis of glucocorticoid-response transcripts in the PVN of male rats after long term high-dose corticosterone exposure and found suppression of *Crf* and *Avp* gene expression, downregulation of the *apelin receptor,* and upregulation of *dual-specificity protein phopsphatase 1* (*Dusp1*). These changes may contribute to the suppression of *Crf* and *Avp* gene expression [104]. In particular, *Dusp1* encodes a phosphatase that inactivates the MAPK. Glucocorticoids-induced *Dusp1* upregulation might counteract MAMP-mediated *Crf* gene expression. Immediate early genes, such as the *brain-specific homeobox protein homolog*, *early growth-response protein 1, Fos*, *Fosb*, *Junb*, and *nuclear receptor subfamily 4 group A member 3,* were transiently upregulated by acute corticosterone administration, and may participate in transcriptional modulation of glucocorticoids target genes.

## 6. Conclusions

In summary, various stressors can activate the HPA axis. CRF, a key player in stress responses, is produced in the PVN in response to stress and plays an important role in the stress response as main initiator of the HPA axis. The stress response also activates a physiological system for stress adaptation. The activated the HPA axis is suppressed by the negative feedback effect of glucocorticoids. Glucocorticoids are essential for stress resilience. ICER can suppress the stress response via inhibition of the cAMP-dependent CRF gene. SOCS-3, stimulated by IL-6 and cAMP, is also involved in the negative regulation of *Crf* gene expression in the hypothalamus. The CRF_1_ receptor is mainly involved in stress responses, while the CRF_2_ receptor mediates “stress coping”, for which glucocorticoids are essential. The differential effects of FKBP4 and FKBP5 further contribute to glucocorticoid efficiency under stress resilience. Altogether, a variety of factors contribute to stress resilience. Importance of each factor, however, remains to be explored. The differential roles of all these factors under stress resilience would be elucidated in the future.

## Data Availability

Data are contained within the article.
